# Genome-Wide Association Study and Meta-Analysis Uncovers Key Candidate Genes for Body Weight Traits in Chickens

**DOI:** 10.3390/genes16080945

**Published:** 2025-08-11

**Authors:** Jintian Wen, Ming Zheng, Zhaochuan Wang, Xiaoxiang Hu, Zhenhui Li

**Affiliations:** 1 State Key Laboratory of Swine and Poultry Breeding Industry, South China Agricultural University, Guangzhou 510642, China; wenjintian@stu.scau.edu.cn (J.W.); zhengming@gzgs.edu.cn (M.Z.); zuisc@stu.scau.edu.cn (Z.W.); 2 Guangdong Laboratory for Lingnan Modern Agriculture, College of Animal Science, South China Agricultural University, Guangzhou 510642, China; 3 Guangdong Provincial Key Lab of Agro-Animal Genomics and Molecular Breeding, Key Laboratory of Chicken Genetics, Breeding and Reproduction, Ministry of Agriculture and Rural Affair, South China Agricultural University, Guangzhou 510642, China; 4 State Key Laboratory of Agrobiotechnology, College of Biological Sciences, China Agricultural University, Beijing 100193, China; huxx@cau.edu.cn

**Keywords:** chicken, body weight traits, GWAS, meta-analysis

## Abstract

Background: Genome-wide association studies (GWAS) have been extensively employed to elucidate the genetic architecture of body weight (BW) traits in chickens, which represent key economic indicators in broiler production. With the growing availability of genomic data from diverse commercial and resource chicken populations, a critical challenge lies in how to effectively integrate these datasets to enhance sample size and thereby improve the statistical power for detecting genetic variants associated with complex traits. Methods: In this study, we performed a multi-population GWAS meta-analysis on BW traits across three genetically distinct chicken populations, focusing on BW at 56, 70, and 84 days of age: P1 (N301 Yellow Plumage Dwarf Chicken Line; *n* = 426), P2 (F2 reciprocal cross: High Quality Line A × Huiyang Bearded chicken; *n* = 494), and P3 (F2 cross: Black-bone chicken × White Plymouth Rock; *n* = 223). Results: Compared to single-population GWAS, our meta-analysis identified 77 novel independent variants significantly associated with BW traits, while gene-based association analysis implicated 59 relevant candidate genes. Functional annotation of BW56- and BW84-associated SNPs (single-nucleotide polymorphisms) 1_170526144G>T and 1_170642110A>G, integrated with tissue-specific regulatory annotations, revealed significant enrichment of enhancer and promoter elements for *KPNA3* and *CAB39L* in muscle, adipose, and intestinal tissues. Through this meta-analysis and integrative genomics approach, we identified novel candidate genes associated with body weight traits in chickens. Conclusions: These findings provide valuable mechanistic insights into the genetic mechanisms underlying body weight regulation in poultry and offer important references for selective breeding strategies aimed at improving production efficiency in the poultry industry.

## 1. Introduction

Poultry production constitutes a significant global agricultural sector, with poultry meat and eggs ranking among the most extensively consumed animal-derived protein sources internationally. Body weight is a fundamental production trait in broilers, profoundly influencing meat yield, economic returns, and key efficiency metrics such as slaughter yield and feed conversion ratio [1,2]. Enhancing the genetic potential for body weight is therefore imperative for optimizing productivity and ensuring sustainable poultry breeding [3]. Genome-wide association studies (GWAS) have revolutionized genetic research on complex traits, facilitating the identification of loci associated with body weight in chickens. These studies provide crucial insights into the genetic determinants of body weight variations across breeds and enable the discovery of novel molecular markers that can accelerate breeding programs [4,5,6,7,8]. However, the substantial cost associated with high-density genotyping frequently necessitates that GWAS for economically important traits in livestock be conducted within single populations of limited sample size. This constraint inevitably results in diminished statistical power for association detection.

Meta-analysis of GWAS data represents a powerful approach for increasing statistical power, minimizing false positives, and elucidating genetic architectures underlying complex traits [9]. In human genetics, Sniekers et al. [10] conducted a GWAS meta-analysis on cognitive traits involving 78,308 individuals, identifying 18 significant genomic regions associated with intelligence. Similarly, in livestock research, Bouwman et al. [11] performed a meta-analysis on height-related traits in cattle, identifying 163 significant genomic loci across 17 populations. The application of GWAS meta-analysis extends to swine genetics, exemplified by Guo et al. [12], who identified key genes associated with limb bone lengths in pigs. Furthermore, Xu et al. [13] executed a comprehensive GWAS meta-analysis of 232 complex porcine traits, revealing the genetic variation rs344053754 to be associated with litter weight at weaning. In poultry research, Ma et al. [14] leveraged genomic data from 6842 broiler breeders in a GWAS meta-analysis, demonstrating that loci on chromosomes GGA4 (encompassing the *AR/YIPF6/STARD8* gene) and GGA7/19/27 co-regulate egg weight and age at first egg via distinct genetic mechanisms, thereby highlighting their pivotal roles in modulating reproductive performance. Liu et al. [15] integrated GWAS meta-analysis with transcriptomic data to identify key regulatory genes (*RAP2C*, *NFKBIA*, *CSF1R*, and *TLR2A*) that influence the relative growth rate (RGR) in white-feathered broilers, mediated through the MAPK signaling pathway and breed-specific expression differences.

The gene-centric association approach, by systematically integrating common genetic polymorphisms within the target gene region and performing joint modeling, can effectively mitigate the inconsistency in association signals caused by population genetic heterogeneity [16]. Gene-based association analysis has been extensively utilized in large-scale human cohort studies, exemplified by prominent initiatives such as the UK Biobank and All of Us Research Program, where systematic gene-level association analyses were conducted using exome sequencing data from 394,841 individuals [17]. Notably, this methodological framework has recently demonstrated translational potential in livestock genomics, evidenced by the identification of eight genes significantly associated with abdominal fat deposition (AFF) through its application in three distinct Yorkshire swine populations [18]. Particularly groundbreaking was the discovery of *PPP1CC*, a pivotal gene regulating meat color phenotype in fast-growing white-feathered broilers, achieved through this analytical paradigm [19]. Despite its proven efficacy in mammalian species, the implementation of gene-based association approaches in avian genomic research remains relatively underexplored, presenting both scientific challenges and opportunities for advancing poultry breeding strategies through precision genomics.

In this study, we conducted a GWAS meta-analysis on body weight traits across three genetically diverse chicken populations. We further performed gene-level association analysis and functional annotation of cross-tissue regulatory elements to identify key SNPs and candidate genes influencing growth traits. Our findings provide novel insights into the genetic foundation of body weight in chickens and hold potential for advancing molecular breeding programs in poultry.

## 2. Materials and Methods

### 2.1. Populations and Phenotypic Data

We analyzed data from three distinct chicken populations, comprising a total of 1143 individuals: P1 (N301 Yellow Plumage Dwarf Chicken Line Population; *n* = 426) [20], P2 (F2 reciprocal cross: High Quality Line A × Huiyang Bearded chicken; *n* = 494) [21], and P3 (a Black-bone chicken × White Plymouth Rock F2 population; *n* = 223) [22]. Body weight measurements were recorded at 56, 70, and 84 days of age. For genotyping, the P1 population was analyzed using the 600 K Affymetrix Axiom HD genotyping array [23], while P2 and P3 were genotyped using the Illumina 60 K SNP Beadchip [24]. SNP coordinates were converted to the GRCg6a genome reference using liftover (http://genome.ucsc.edu/cgi-bin/hgLiftOver (accessed on 5 March 2025)). Quality control was performed using PLINK v1.9 [25], applying the following criteria: individual missing rate < 0.01, site missing rate < 0.01, and minor allele frequency (MAF) > 0.01 [26]. Genotype imputation was conducted using the ChickenGTEx genomic reference panel [27], employing Beagle 5.1 software [28] with filtering parameters of MAF < 0.01 and DR2 < 0.4.

### 2.2. Genetic Parameter Estimation

Genetic parameters were estimated using the Restricted Maximum Likelihood (REML) method with an animal model, implemented in the Hiblup v1.4.0 software [29]. The model equation is formulated as follows:

*y* = 1*μ* + *Xb* + *Za* + *e*

where *y* represents the vector of phenotypic values for growth traits. *μ* is the overall mean. *b* is the vector of fixed effects (including sex and batch). *a* is the vector of additive genetic effects for individuals, where a~*N* (0, *G*$\sigma_{a}^{2}$), with *a*^2^ representing. *G* denotes the genomic relationship matrix. *e* is the vector of residual effects, assumed to be *e*~*N* (0, *I*$\sigma_{e}^{2}$), where *I* is the identity matrix; *X* and *Z* are the design matrices corresponding to *b* and *a*, respectively.

The heritability calculation is as follows:

$$h_{\left(t\right)}^{2}\;=\;\frac{\sigma_{a(t)}^{2}}{\sigma_{a(t)}^{2}+\sigma_{e(t)}^{2}}$$

$\;h_{\left(t\right)\;}^{2}$ represents heritability, $\sigma_{a(t)}^{2}$ represents the additive genetic variance, and $\sigma_{e(t)}^{2}$ represents the residual variance.

The genetic correlation between growth traits is calculated as follows:

$$r_{g}=\frac{{\sigma_{a}^{2}\;}_{(t1,t2)}}{\sqrt{\sigma_{a(t1,t1)}^{2}\times \sigma_{a(t2,t2)}^{2}}}$$

$r_{g}$ represents the genetic correlation, $\sigma_{a(t1,t1)}^{2}$, and $\sigma_{a(t2,t2)}^{2}$ denote the genetic variances of the two traits, and ${\sigma_{a}^{2}\;}_{(t1,t2)}$ corresponds to the genetic covariance between the two traits. The calculation of phenotypic correlation follows a similar approach to that used for genetic correlation.

### 2.3. GWAS Analysis

To identify and correct for population stratification among the test animals, principal component analysis (PCA) was performed using PLINK v1.9. The first five principal components were subsequently incorporated as covariates in all downstream analyses to account for potential population structure.

Genome-wide association analysis for individual populations was conducted using fastGWA from GCTA v1.94 [30]. The analysis employed a linear mixed model formulated as follows:

*y* = *Xa* + *Zb* + *Wg* + *e*

*y* represents the phenotypic values of n individuals in the study population; matrix *X* contains fixed effects and covariates, including sex (fixed effect 1), batch (fixed effect 2), and the first five principal components (covariates); *a* is the vector of corresponding effects; *Z* is the genotype vector for the variant sites; *b* is the effect of the variant sites; *W* is the incidence matrix for random additive effects; *g* is the vector of random additive effects, where *g*~*N* (0, *Gσg*^2^), with *G* being the genomic relationship matrix; and *e* is the vector of random residuals, assumed to be *e*~*N* (0, *Iσe*^2^), where *I* is the identity matrix.

To determine the number of independent genome-wide markers, we employed PLINK v1.9, using the command indep-pairwise 25 5 0.2, with an r^2^ threshold of 0.2. The threshold for genome-wide significance was set based on the number of independent SNPs. Manhattan plots and Q-Q plots were generated using the CMplot package in R v4.3.1 (https://github.com/YinLiLin/CMplot (accessed on 10 March 2025)).

### 2.4. Meta-Analysis

The meta-analysis of genome-wide data has significantly advanced our understanding of complex traits, addressing some of the missing genetic variance that cannot be identified through simple-population association studies [9]. To integrate results from three independent GWAS, we conducted a meta-GWAS using METAL (released on 2020-05-05) demploying the fixed-effect inverse variance weighting method [31].

### 2.5. Definition of Significant Independent Loci

Identification of significant independent loci was performed using the clumping method in PLINK v1.9. The genome-wide significance threshold was defined as 1 divided by the number of independent SNPs. The –clump-r2 parameter was set to 0.01, and –clump-kb was set to 1000, corresponding to a window size of 1 Mb, to determine the index variant sites.

### 2.6. Gene Association Analysis

To further investigate the genetic determinants of body weight traits, we performed GWAS by utilizing SNP-based *p*-values derived from the three meta-analyses as input for gene-level association testing. The chicken genome annotation file Gallus_gallus.GRCg6a.106.gtf.gz (https://ftp.ensembl.org/pub/release-106/gtf/gallus_gallus/Gallus_gallus.GRCg6a.106.gtf.gz (accessed on 15 March 2025)) was used for annotation, and the analysis was performed using MAGMA v1.10 [32].

### 2.7. SNP QTL Annotation

To functionally characterize the identified independent SNPs, we performed quantitative trait loci (QTL) annotation. SNP annotation was conducted using the Variant Effect Predictor (VEP) from Ensembl (http://www.ensembl.org (accessed on 20 March 2025)) [33]. The QTL region was defined as ±0.5 Mb flanking each significant SNP. QTL enrichment analysis was performed using GALLO v1.5 [34], leveraging data from the Animal QTL database [35]. The adjusted *p*-value threshold for significance was set at 0.05, ensuring statistical rigor in the enrichment analysis.

### 2.8. Functional Annotation of Candidate Genes

To further investigate the biological functions of these genes, we performed functional annotation of the candidate genes identified from the meta-analysis (Meta-GWAS) and gene association analysis of the three independent populations. Gene Ontology (GO) term and Kyoto Encyclopedia of Genes and Genomes (KEGG) pathway enrichment analyses were conducted using the KOBAS website [36].

### 2.9. Linkage Disequilibrium and Chromatin State Annotation of Genome-Wide Significant Loci

To further investigate the genetic architecture of significant SNP loci, we conducted linkage disequilibrium (LD) analysis using the locuszoomr v0.3.2 R package, evaluating r^2^ values between SNPs within the candidate regions [37]. Chromatin state annotation was conducted using the UCSC Genome Browser website (http://genome.ucsc.edu/s/zhypan/galGal6_FAANG_V1 (accessed on 24 March 2025)), which provides chromatin state data across 23 tissue types. To visually discriminate chromatin states, distinct regulatory elements were color-coded, facilitating the delineation of functional genomic elements at significant loci associated with body weight traits.

## 3. Results

### 3.1. Phenotypic Statistics and Genetic Parameters for Growth Traits

Descriptive statistics for growth traits across three chicken populations at 56, 70, and 84 days of age are presented in Table S1. The coefficients of variation (CV) for body weight (BW) in the three populations were 13.726–17.995%, 11.807–17.503%, and 11.892–17.549%, respectively, indicating significant phenotypic variation in BW traits among individuals within populations of distinct genetic backgrounds. Notably, the P3 population exhibited more pronounced differences across the different BW traits. To estimate heritability at the three time points, single-trait analyses were performed using HIBLUP. The heritability estimates for BW at 56 days of age (BW56) ranged from 0.36 to 0.66, for BW at 70 days (BW70) from 0.31 to 0.64, and for BW at 84 days (BW84) from 0.32 to 0.63, demonstrating moderate to high heritability levels (Table S1). For genetic correlations, strong positive correlations were observed among all BW traits (rg 0.839~0.998). Notably, the P2 population displayed consistently higher heritability across all three age points. Importantly, the strong positive correlations among all BW traits suggest shared genetic influences across different growth stages (Table S2).

This section may be divided by subheadings. It should provide a concise and precise description of the experimental results and their interpretation, as well as the experimental conclusions that can be drawn.

### 3.2. Population Structure

**Figure 1:**
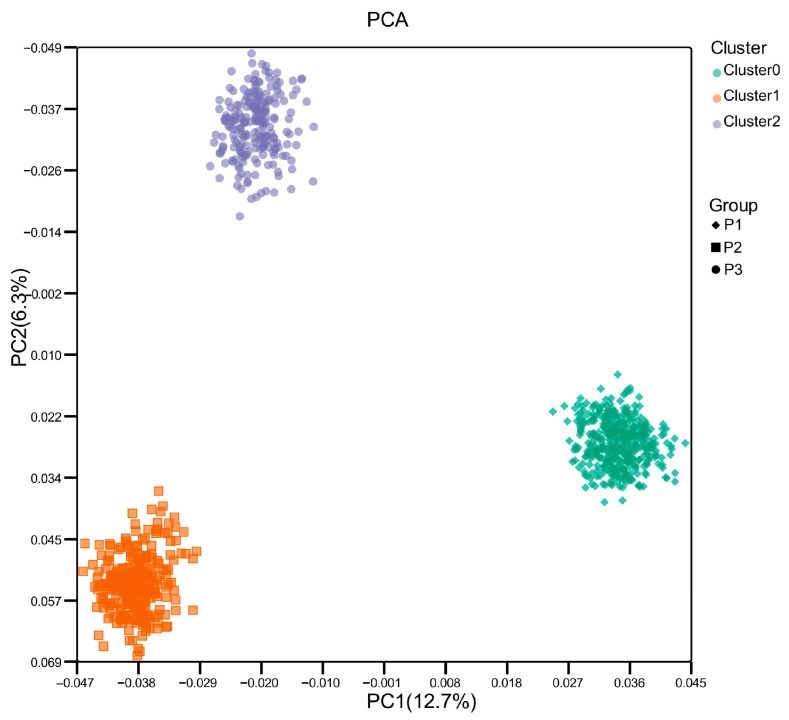
Principal Component Analysis (PCA) of population structure showing the top two principal components. PC1: Principal component 1; PC2: Principal component 2. The green dot represents the P1 (N301 Yellow Plumage Dwarf Chicken Line Population); the orange dot represents the P2 (F2 reciprocal cross: High Quality Line A × Huiyang Bearded chicken); the purple dot represents the P3 (a Black-bone chicken × White Plymouth Rock F2 population).

To comprehensively analyze the genetic relationships among the three populations, we performed principal component analysis (PCA) utilizing genome-wide SNP array data. The PCA visualization demonstrated clearly delineated and well-separated clusters corresponding to each population, indicating pronounced genetic divergence among these groups (Figure 1). To mitigate potential confounding from population stratification, the first five principal components (PCs), collectively explaining 24.1% of the genetic variance, were incorporated as covariates within the genome-wide association study model.

### 3.3. Single-Population GWAS Results

**Figure 2:**
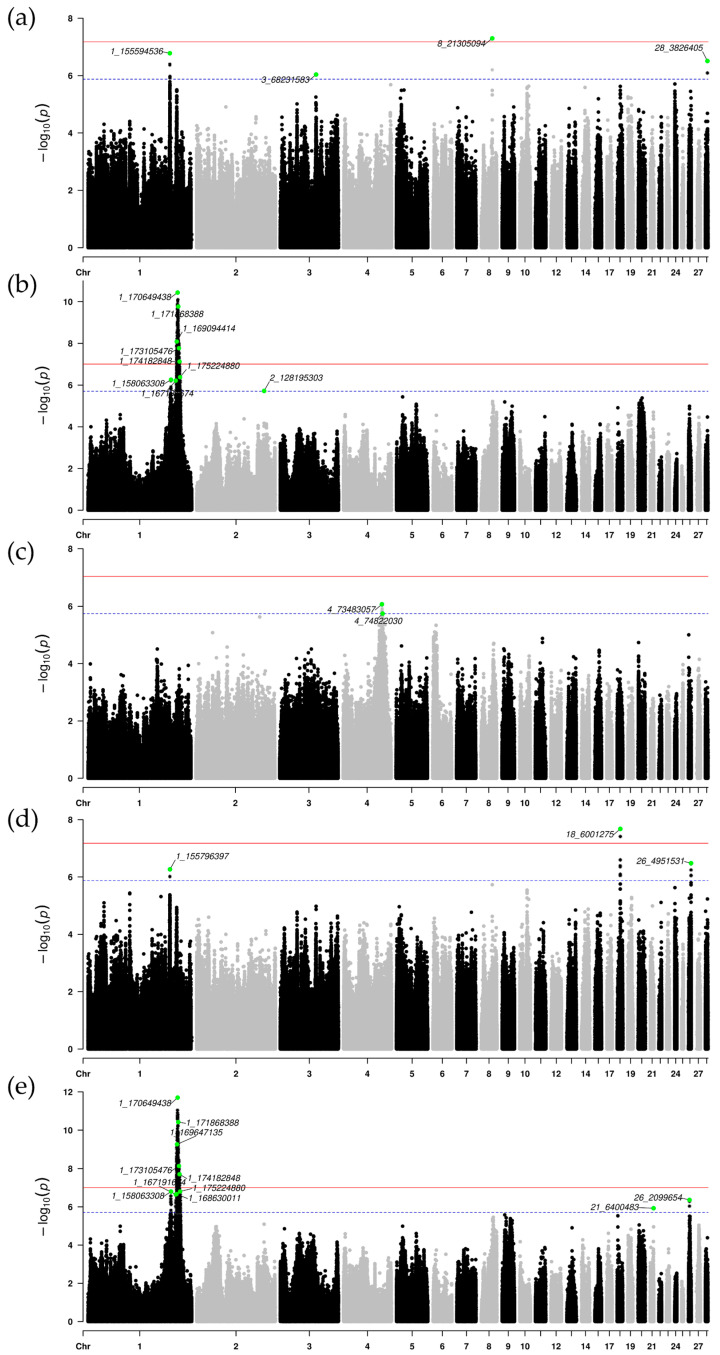

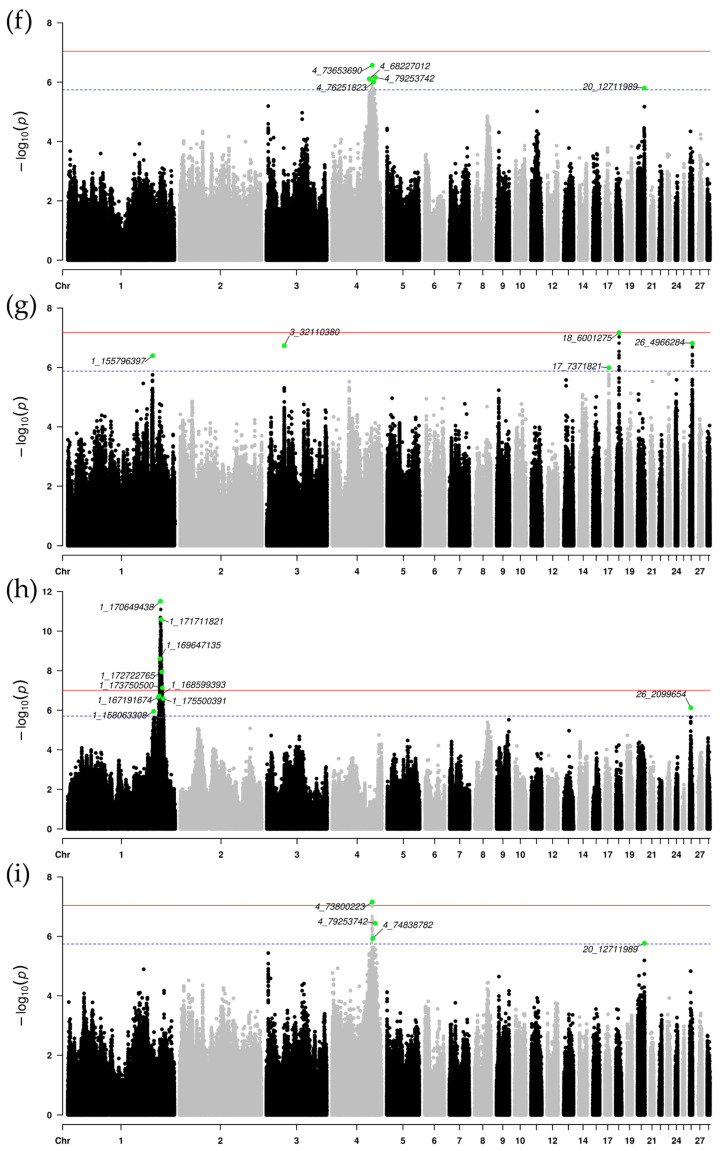
Manhattan plots of GWAS analysis for growth traits across three chicken resource populations. Each point represents an individual SNP. Panels (**a**–**c**) display the 56-day body weight for populations P1, P2, and P3, respectively. Panels (**d**–**f**) represent the 70-day body weight for populations P1, P2, and P3, while panels (**g**–**i**) depict the 84-day body weight for the same populations. Independent significant SNPs are indicated by green circular markers. The red and blue horizontal lines represent the genome-wide significance threshold and the suggestive significance threshold, respectively.

Following the completion of genotype imputation, the final number of SNP loci identified across the three populations was as follows: P1 (8,688,718), P2 (7,020,357), and P3 (6,304,433). Subsequently, independent genome-wide association studies (GWAS) were conducted on each of these three populations to analyze their respective genetic associations (Table S3). Using a suggestive significance threshold (1/N), independent variants associated with growth traits were identified. The detection of independent loci enhances the accuracy and reliability of GWAS analysis by reducing redundant signals caused by linkage disequilibrium (LD), thereby enabling the precise identification of key variant loci associated with traits [38]. The PLINK v1.9 clumping method identified 10, 18, and 9 independent SNPs associated with growth traits in each population, respectively (Table S4). Of these 37 identified SNPs, 3 were located in intergenic regions, 22 in intronic regions, 6 upstream of coding sequences, 5 in downstream regions, and 1 in the 5′ untranslated region (5′UTR). 15 SNPs showed significant association with BW56, 19 SNPs were significantly associated with BW70, and 19 SNPs demonstrated significant association with BW84. The highest concentration of significant SNPs was observed on chromosomes GGA1 (17 SNPs) and GGA4 (8 SNPs) (Figure 2). The QTL enrichment analysis conducted using the Animal QTL database revealed significant enrichment of SNPs associated with key growth, carcass, and feed efficiency traits, including body weight, gizzard weight, proventriculus weight, and feed intake. Furthermore, the analysis also found that these SNPs overlap with QTLs for important reproductive and egg quality traits, such as egg weight, yolk weight, ovary weight, and oviduct length (Table S5). This overlap may indicate a genetic correlation between growth traits and reproductive traits. These findings underscore the genetic basis of growth-related traits in chickens.

### 3.4. Meta-Analysis of GWAS Results

**Figure 3:**
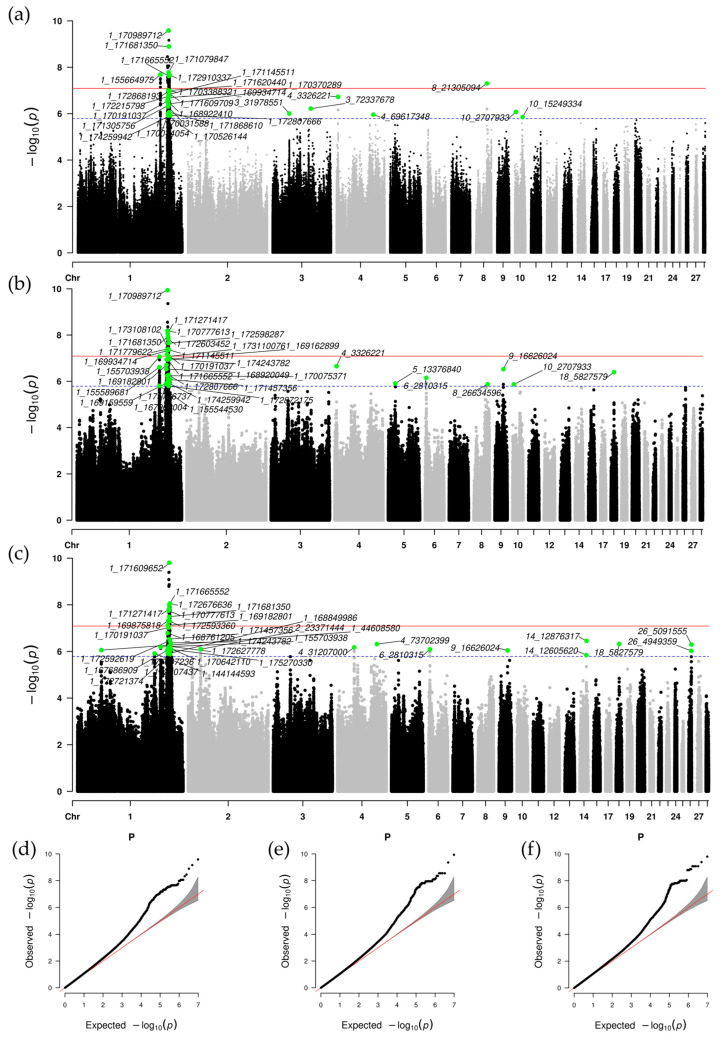
Manhattan plots and Q-Q plots for the meta-analysis of three growth traits. Panels (**a**–**c**) show the Manhattan plots for body weight at 56 days of age (BW56), 70 days of age (BW70), and 84 days of age (BW84), respectively. Green circular dots represent independent significant SNPs. The red and blue horizontal lines correspond to the genome-wide significance threshold and the suggestive significance threshold, respectively. Panels (**d**–**f**) display the corresponding Q-Q plots for each trait.

To enhance detection power and accuracy, a meta-analysis was performed across the three populations. Utilizing the PLINK clumping method, we identified 31, 35, and 34 significant SNPs associated with body weight at BW56, BW70, and BW84, respectively, resulting in a total of 78 loci (Figure 3). These SNPs are distributed across chromosomes GGA1-6, 8–10, 14, 18, and 26. Due to the distinct genetic backgrounds of the three populations, a meta-analysis was conducted. Compared to single-population GWAS results, the meta-analysis identified an additional 77 new SNPs, significantly enhancing the discovery of novel SNPs. Notably, chromosomes GGA1 (with 59 SNPs) and GGA4 (with 4 SNPs) exhibited the highest enrichment. Furthermore, compared to single-population GWAS, the meta-GWAS revealed a markedly greater number of significant loci on chromosome 1 (Figure S1a). The QQ plots for all growth traits (Figure 3d–f) showed genomic inflation factors (λ) of 1.079 (BW56), 1.048 (BW70), and 1.067 (BW84), demonstrating adequate control of population structure. We annotated the candidate genes through independent variant annotation and performed functional annotation, with 28, 30, and 33 candidate genes annotated for the three traits’ SNPs (Table S6). In addition, the QTL enrichment analysis indicated that the QTL characteristics derived from the integrated analysis encompassed important production traits such as body weight, breast muscle weight, average daily gain, and feed intake (Table S7). Compared to the enrichment results of individual population GWAS, interestingly, we identified new QTLs (breast muscle weight, subcutaneous fat thickness, and white striping) that overlapped with SNPs from the meta-analysis. Among these, the white striping trait is closely associated with high feed efficiency and rapid growth in broilers, particularly breast muscle development [39]. A total of 28 GO terms (top 10 ranked by enrichment score, *p*-value < 0.05) and KEGG pathways (*p*-value < 0.05) were identified. GO analysis showed that candidate genes were significantly enriched in the citric acid cycle (gga00020) and N-glycan biosynthesis (gga00510), while KEGG analysis revealed enrichment in axonogenesis of central nervous system projection neurons and negative regulation of amyloid precursor protein biosynthesis (Table S9).

### 3.5. Gene Association Analysis

**Figure 4:**
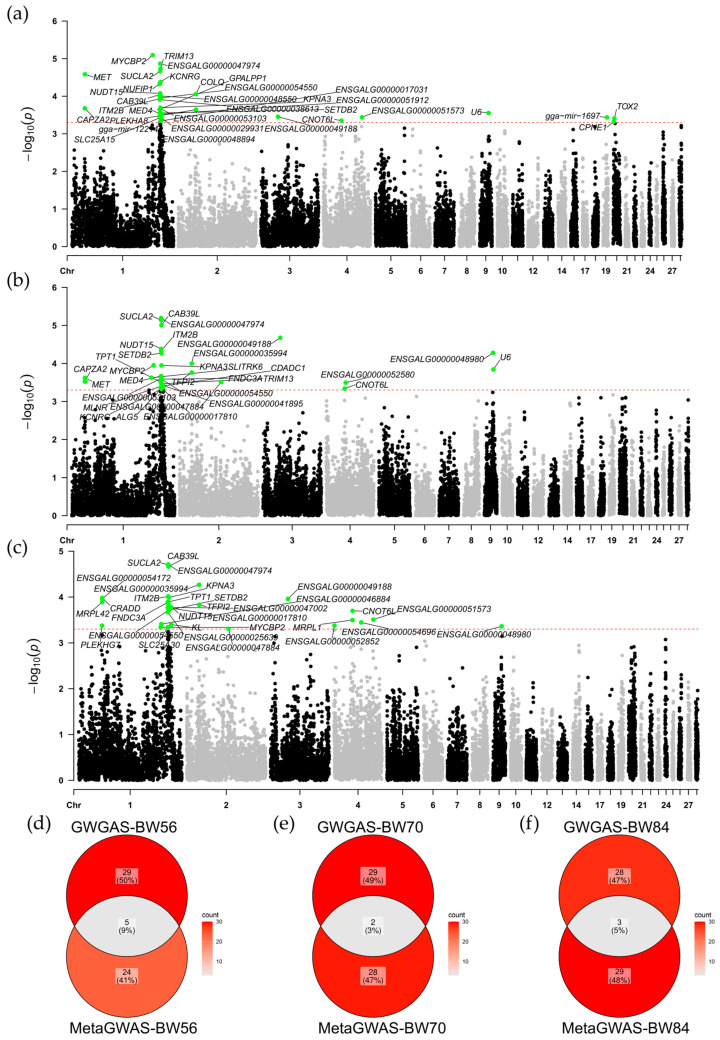
Manhattan plots for the genome-wide gene association studies (GWGAS) of three growth traits. Panels (**a**–**c**) display the Manhattan plots for body weight at 56 days of age (BW56), 70 days of age (BW70), and 84 days of age (BW84), respectively. The negative log10 transformation of the *p*-values for each gene is plotted. Green dots represent genes that showed significant associations in the GWGAS. The red dashed line indicates the significance threshold (*p* = 0.0005). Panels (**d**–**f**) show Venn diagrams illustrating the overlap of genes between Meta-GWAS and GWGAS for BW56 (**d**), BW70 (**e**), and BW84 (**f**).

A genome-wide gene association study (GWGAS) was conducted using MAGMA v1.10, enabling the identification of novel gene-level associations that may not be captured by SNP-based GWAS. Out of 19,959 genes analyzed, significant associations were identified for 34 genes linked to BW56, 31 genes linked to BW70, and 31 genes linked to BW84. The candidate genes were predominantly localized on chromosome 1, with 25 (74%), 23 (74%), and 21 (68%) genes associated with each of the traits, respectively. The remaining candidate genes were distributed across chromosomes 2, 4, 9, and 20 (Figure 4a–c). The Venn diagram illustrates the overlap between GWGAS-identified genes and Meta-GWAS candidate genes, with five (*SETDB2*, *SUCLA2*, *NUFIP1*, *MYCBP2*, *ITM2B*), two (*SUCLA2*, *MYCBP2*), and three (*ENSGALG00000035994*, *KPNA3*, *MYCBP2*) overlapping genes, respectively (Figure 4d–f). Functional annotation identified 27 significantly enriched terms, including the top 10 ranked GO terms (*p*-value < 0.05) and KEGG pathways meeting the significance threshold (*p*-value < 0.05). GO analysis revealed significant enrichment of candidate genes in the citric acid cycle (gga00020) and propanoate metabolism (gga00640). KEGG analysis showed significant enrichment in processes such as axonogenesis in central nervous system projection neurons and the response to reactive oxygen species (Table S10).

### 3.6. Chromatin State Annotation of Genome-Wide Significant Loci

**Figure 5:**
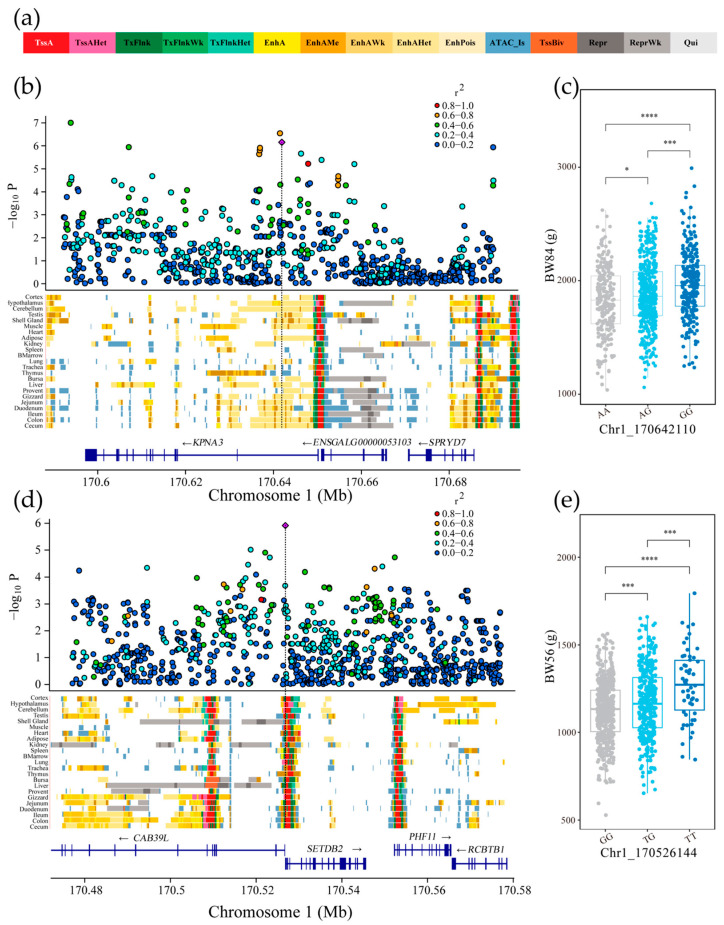
Chromatin state plots for significant independent genomic loci SNPs. (**a**) Fifteen predicted chromatin states. (**b**,**d**) The upper panels display the linkage disequilibrium (LD) plots of the main effect SNPs (Chr1_170642110 A>G) and (Chr1_170526144 G>T) with surrounding SNPs, while the lower panels show the chromatin states surrounding the corresponding significant SNPs. The regions shown extend ±10 kb from the main effect SNPs. Gene distribution within the region is displayed at the bottom of the figure. (**c**,**e**) Phenotypic comparisons between different genotypes based on the significant SNPs (Chr1_170642110 A>G) and (Chr1_170526144 G>T). The X-axis represents the different genotypes, and the Y-axis shows body weight at 56 days of age (BW56) and 84 days of age (BW84). The effects of SNPs on BW56 and BW84 were assessed using *t*-tests. Statistical key: **** *p* < 0.0001; *** *p* < 0.001; * *p* < 0.05.

By integrating the findings from meta-GWAS and GWGAS analyses, we selected candidate genes identified in the intersection of both analyses (*SETDB2*, *KPNA3*) and focused on their independent SNPs, specifically Chr1_170642110 A>G (BW84) and Chr1_170526144 G>T (BW56). Utilizing predicted chromatin state maps, we performed functional annotation of the regulatory elements associated with these independent SNPs (Figure 5a). The results indicate that the Chr1_170642110 site is located within enhancer elements in muscle, adipose, proventriculus, gizzard, and duodenum tissues. This variant is situated in the intron region of the *KPNA3* gene and may play a regulatory role in the tissue-specific expression of *KPNA3* in muscle, proventriculus, adipose, gizzard, and duodenum tissues. Through its coordinated effects across multiple tissues and organs, it influences the overall trait of body weight (Figure 5b and Figure S2a). Individuals carrying the Chr1_170642110 G allele exhibit significantly higher body weight at 84 days of age (Figure 5c).

The Chr1_170526144 site is located in promoter elements in muscle tissue, enhancer elements in proventriculus tissue, and transcription start site (TSS)-proximal transcription region elements in fat, gizzard, and duodenum tissues (Figure 5d and Figure S2b). These tissues play crucial roles in body weight traits by influencing the formation and variation of body weight through mechanisms such as growth, digestion, and nutrient absorption. Individuals carrying the Chr1_170526144 T allele exhibit significantly higher body weight at 56 days of age (Figure 5e).

## 4. Discussion

Body weight is a complex quantitative trait influenced by both genetic and environmental factors. GWAS have been extensively utilized to investigate the genetic architecture of growth-related traits across various species. In humans, over 1000 genetic loci associated with body weight and obesity have been identified, elucidating key biological pathways involved in energy metabolism, appetite regulation, and fat distribution [40,41]. In livestock breeding, body weight is a critical economic trait in pigs and cattle, and its genetic basis has been widely explored. For instance, a 60 K SNP chip-based GWAS identified *TBC1D1*, *BAAT*, and *PHLPP1* as key candidate genes associated with carcass weight and backfat thickness in pigs through single-trait GWAS, multi-trait GWAS, and meta-analysis methods [42]. Similarly, in cattle populations from the Russian Federation (Siberian region), a 150 K SNP chip GWAS revealed that *CCND2*, located on chromosome 5, was significantly associated with body weight traits [43]. Moreover, a genome-wide association study (GWAS) conducted on Wenshang Barred chickens (*n* = 596) alongside three additional breeds exhibiting varying body sizes—Recessive White (RW), Wenshang Barred (WB), and Luxi Mini (LM) chickens (*n* = 50)—identified significant associations between body weight and body size conformation traits on chromosomes 1 and 4 [8]. Zhong et al. [7] conducted a multidimensional GWAS on chicken body weight (BW) traits, revealing that BW-associated genetic variants exhibited an orderly distribution pattern across three developmental stages. Chromosomes 1 and 4 were identified as exerting pivotal regulatory roles on BW during the first phase (1–7 weeks of age) and the second phase (8–22 weeks of age), respectively. Notably, some of these associations overlapped with the significant genomic intervals identified in our study. Our research findings demonstrated considerable consistency with previous literature, particularly regarding chromosomal distribution patterns. The GWAS analysis conducted across three chicken populations, followed by meta-analysis, revealed that significant SNPs associated with body weight were predominantly concentrated on GGA1 (17 SNPs in individual GWAS, 59 SNPs in meta-analysis) and GGA4 (8 SNPs in individual GWAS, 4 SNPs in meta-analysis). This aligns strongly with findings by [8] and Zhong et al. [7], who similarly identified chromosomes 1 and 4 as playing crucial regulatory roles in chicken body weight, demonstrated that chromosome 1 exerts key functions during early growth stages (1–7 weeks of age), while chromosome 4 becomes more significant during intermediate growth stages (8–22 weeks of age). This corresponds with the SNP distribution patterns we observed across different growth stages (BW56, BW70, and BW84), suggesting temporal specificity in the regulatory functions of these chromosomes during chicken growth and development.

With the advent of whole-genome sequencing (WGS), researchers have gained powerful tools to uncover genetic variations associated with complex traits in animal breeding [44,45]. However, despite its advantages, high-coverage WGS data remain relatively scarce in practical breeding applications due to cost constraints. As a result, genotype imputation, which infers high-density genotypes from low-density genotyping arrays or low-coverage sequencing data, has become a widely adopted strategy in GWAS [28,46,47]. This approach offers multiple benefits, including cost efficiency, enhanced accuracy, and improved genomic resolution, making it an indispensable tool in genetic studies. Several poultry-specific genomic reference panels have been established to facilitate genotype imputation [27,48,49]. In this study, we utilized the ChickenGTEx genomic resequencing reference panel to impute 55 K and 600 K SNP chip data, enabling a more comprehensive association analysis of body weight traits across three genetically diverse chicken populations. The effectiveness of this strategy was fully demonstrated in our research results. After genotype imputation, we identified a substantial number of SNP loci across the three populations: P1 (8,688,718), P2 (7,020,357), and P3 (6,304,433), indicating that the ChickenGTEx reference panel can significantly enhance genomic coverage and resolution. Compared to using only original chip data, this high-density marker approach considerably improved our ability to discover genetic variations associated with growth traits.

The meta-analysis GWAS approach significantly enhances statistical power by integrating genetic signals across populations [50]. Systematic investigations employing meta-analytic GWAS approaches for elucidating the genetic architecture of avian body weight traits across diverse populations remain notably limited. In our study, meta-analysis GWAS were performed, leading to the identification of 78 independent significant SNPs, among which 77 were uniquely detected through meta-analysis. In genomic selection, the integration of prior information on single-nucleotide polymorphisms obtained from GWAS meta-analyses enhances predictive performance [51]. This methodological advancement can be seamlessly incorporated into our research framework, thereby facilitating the prediction of economically significant traits in poultry. Unlike previous avian studies, we employed GWAS summary statistics in conjunction with the PLINK clumping method to refine the selection of independent loci. Furthermore, we conducted fine-mapping of these loci to assess their associations with growth traits at higher resolution. Through meta-GWAS and gene association analyses, we identified three candidate SNP loci and their associated genes linked to BW traits. Functional enrichment analysis demonstrated that these genes are predominantly involved in pathways such as the citric acid cycle (gga00020), N-Glycan biosynthesis (gga00510), propanoate metabolism (gga00640), and the negative regulation of central nervous system projection neuron axonogenesis and amyloid precursor protein biosynthesis.

Pan et al. [52] identified 15 different chromatin states across 23 tissues, including TssA (strong active promoter/transcription region), TssAHet (active promoter flanking region without ATAC), TxFlnk (gene body transcription region), TxFlnkWk (weak gene body transcription region), TxFlnkHet (transcription region without ATAC), EnhA (strong active enhancer), EnhAMe (moderate active enhancer with ATAC), EnhAWk (weak active enhancer), EnhAHet (active enhancer without ATAC, heterochromatin), EnhPois (pre-promoter enhancer), ATAC_Is (ATAC island), TssBiv (bivalent/pre-activated promoter), Repr (repressed Polycomb region), ReprWk (weakly repressed Polycomb region), and Qui (quiescent region) (Figure 5a). Combining GWAS results with cross-tissue regulatory element functional annotations provides a more comprehensive understanding of the role of SNPs in gene expression regulation, particularly their tissue-specific differences, revealing the genetic mechanisms influencing production traits. Functional annotation of the regulatory elements associated with the significant SNPs implicated *KPNA3*, *CAB39L*, and *SETDB2* as key regulatory genes. These loci exhibited strong associations with tissue-specific enhancers and promoter elements in muscle, adipose, and intestinal tissue, indicating their potential regulatory roles in body weight determination. This finding is consistent with previous research, which identified the *KPNA3* gene as a candidate gene influencing breast muscle weight through genome-wide association analysis [53]. We found that this variant site is located upstream of the *CAB39L* and *SETDB2* genes, which may have a regulatory role in the specific expression of these two genes in muscle, proventriculus, fat, gizzard, and duodenum tissues, thereby playing an important role in growth and body weight gain [54]. Previous studies have identified *KPNA3* as a critical gene for muscle growth in cattle, and GWAS analysis in Wen Shang Luhua chickens also revealed significant associations between *KPNA3* and growth-related traits [55]. Moreover, in Ghanaian local chicken, GWAS analysis identified *CAB39L* and *SETDB2* as being associated with carcass and internal organ traits [56]. ATAC-seq analysis across 11 chicken tissues confirmed *CAB39L* as actively transcribed in duodenal tissue [57]. The Calcium Binding Protein 39-Like (*CAB39L*) gene, located within the 169.025–169.086 Mb region of Chromosome 1, participates in the mTOR signaling pathway. The SET Domain Bifurcated 2 (*SETDB2*) gene, located within the 169.087–169.105 Mb region of Chromosome 1, participates in the Lysine Degradation signaling pathway. These two genes mediate the enrichment of their expression products in the duodenum through their respective mTOR and Lysine Degradation signaling pathways, thereby regulating body weight and growth/development. Overall, the integration of GWAS, meta-GWAS, gene association analysis, and regulatory element annotation provides deeper insights into the molecular mechanisms underlying growth traits in chickens. These findings not only contribute to the fundamental understanding of avian genetics but also offer valuable genomic resources for poultry breeding programs, facilitating the development of precision breeding strategies aimed at enhancing economic traits.

Although the current study conducted meta-GWAS analyses for BW56, BW70, and BW84, it is noteworthy that chicken body weight development is a continuous, long-term process. Incorporating phenotypic and genomic data from additional time points would facilitate more effective identification of both temporally specific and persistently acting genetic loci [7]. Research has demonstrated that the statistical power of genome-wide association studies is positively correlated with sample size, with detection capability significantly enhanced as study scale increases [58,59]. In our research, further expansion of the sample size is necessary to identify rarer variants associated with body weight. Distinct populations exhibit heterogeneous genetic architectures and population-specific linkage disequilibrium (LD) landscapes, potentially leading to the dilution or omission of loci demonstrating significant allele effect sizes within isolated cohorts during meta-analytic integration [60,61]. Despite rigorous quality control protocols and principal component-based adjustments for population stratification applied to individual cohorts, residual heterogeneity inherent in cross-population genetic differentiation may still confound meta-analysis effect estimates. Future research ought to prioritize the aggregation of ancestrally diverse cohorts and implement advanced statistical frameworks, such as hierarchical models incorporating genome-derived differentiation matrices or meta-regression approaches explicitly modeling cross-population heterogeneity covariates [62].

## 5. Conclusions

In this study, we conducted a comprehensive genome-wide association study meta-analysis by integrating imputed SNP chip data from three genetically distinct chicken populations. This analysis identified 30, 35, and 35 significant independent candidate single-nucleotide polymorphisms (SNPs) associated with body weight at 56, 70, and 84 days of age (BW56, BW70, and BW84), respectively. Subsequent gene association analysis revealed 34 (BW56), 31 (BW70), and 31 (BW84) significantly associated genes, reinforcing the polygenic nature of body weight regulation in chickens. Furthermore, functional annotation of regulatory elements linked to these SNPs demonstrated their tissue-specific enhancer and promoter activities in muscle, adipose, and intestinal tissues. These newly discovered significant loci, combined with regulatory element annotations, serve as potential targets for molecular breeding. Collectively, our findings offer novel insights into the developmental regulation of body weight traits and establish a robust foundation for genomic selection and molecular breeding programs.

## Data Availability

The data presented in this study are available on request from the corresponding author. The data are not publicly available due to privacy restrictions and the long extension of datasets.

## Supplementary Materials

The following supporting information can be downloaded at https://www.mdpi.com/article/10.3390/genes16080945/s1, Figure S1: (a) UpSet plot of significant GWAS and meta-GWAS loci for individual body weight traits across three chicken resource populations. (b) UpSet plot of candidate genes at significant loci from genome-wide association study (GWAS) and cross-population meta-GWAS for individual body weight traits in three chicken resource populations; Figure S2: Predicted chromatin states for the Chr1_170642110 A>G and Chr1_170526144 G>T loci; Table S1: The Descriptive Statistics of the Body Weight Trait; Table S2: The Phenotypic and Genetic Correlations Among Body Weight Traits; Table S3: Basic Statistics of GWAS Analysis for Three Chicken Populations; Table S4: Information on Independent Variants for Three Individual GWAS of Body Weight Traits; Table S5: QTL Enrichment Results for Significant SNPs Associated with Body Weight Traits in Three Chicken Populations; Table S6: Information on Independent Variants in Meta-GWAS of Body Weight Traits; Table S7: QTL Enrichment Results for Significant SNPs from the Meta-GWAS of Body Weight Traits in Three Chicken Populations; Table S8: List of genes significantly associated in gene-based association analysis; Table S9: KEGG Pathway Enrichment Analysis and GO Annotation of Candidate Genes from Meta-GWAS; Table S10: Enrichment analysis of KEGG pathways and GO annotation for candidate genes from gene association analysis.

## Abbreviations

The following abbreviations are used in this manuscript:

GWAS	Genome-wide association study
GWGAS	Genome-wide gene association analysis
PCA	Principal component analysis
SNPs	Single-nucleotide polymorphisms
BW56	Weight at 56 days
BW70	Weight at 70 days
BW84	Weight at 84 days
