# Supplementary figures and images for: Genome-Wide Association Study and Meta-Analysis Uncovers Key Candidate Genes for Body Weight Traits in Chickens
Source: Genes (Basel). 2025 Aug 11;16(8):945. doi: 10.3390/genes16080945 (PMC12385673; doi:10.3390/genes16080945)

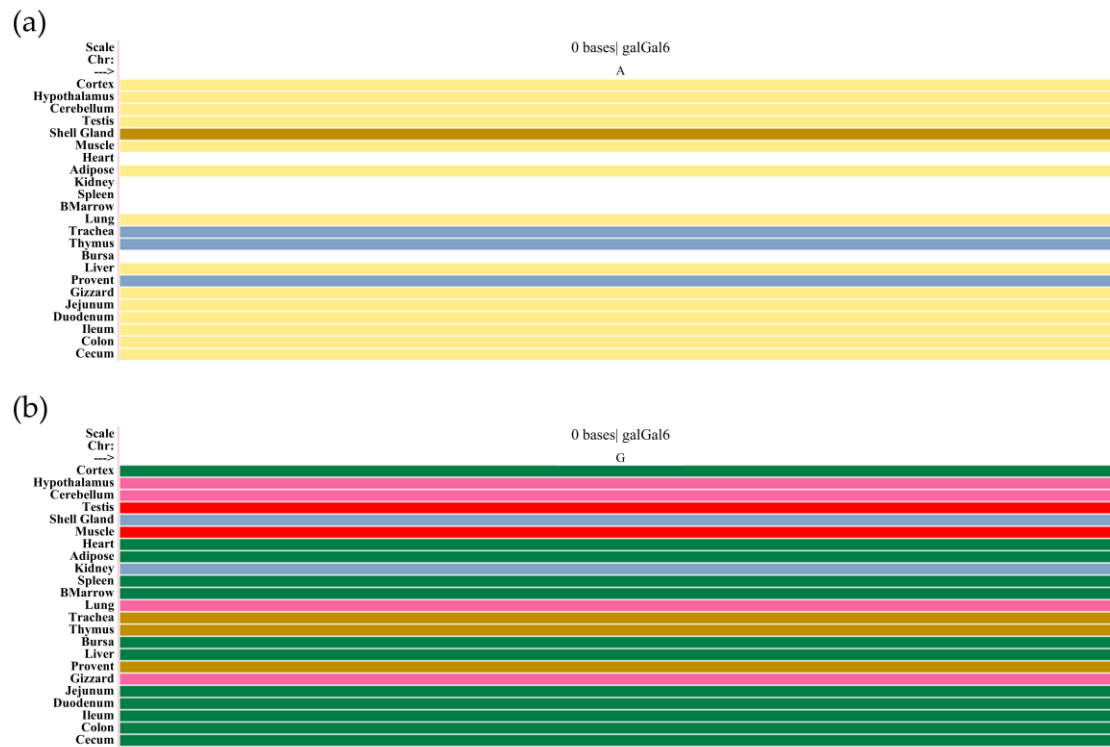

**Figure S2.** Predicted chromatin states for the Chr1\_170642110 A>G and Chr1\_170526144 G>T loci.

Supplement: Supplementary file 1 [file genes-16-00945-s001.zip › Figure_S2.pdf]
