# Supplementary material for: Genome-Wide Association Study and Meta-Analysis Uncovers Key Candidate Genes for Body Weight Traits in Chickens
Source: Genes (Basel). 2025 Aug 11;16(8):945. doi: 10.3390/genes16080945 (PMC12385673; doi:10.3390/genes16080945)
Supplement: Supplementary file 1 [file Figure_S1.pdf › Figure_S1.pdf]

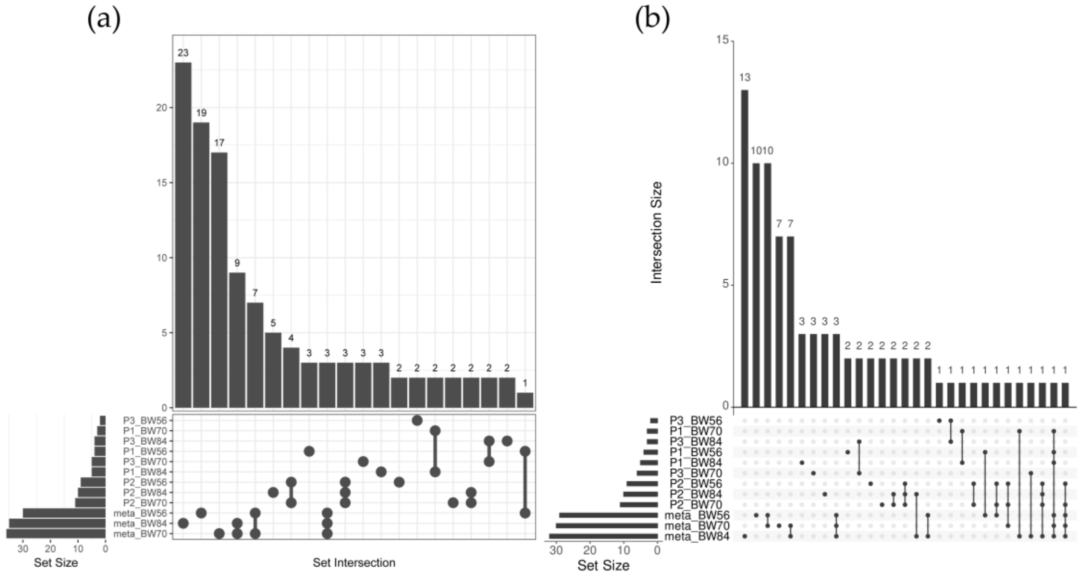

Figure S1. (a) UpSet plot of significant GWAS and meta-GWAS loci for individual body weight traits across three chicken resource populations. (b) UpSet plot of candidate genes at significant loci from genome-wide association study (GWAS) and cross-population meta-GWAS for individu al body weight traits in three chicken resource populations.
